# Quantitative Proteomics and Relative Enzymatic Activities Reveal Different Mechanisms in Two Peanut Cultivars (*Arachis hypogaea* L.) Under Waterlogging Conditions

**DOI:** 10.3389/fpls.2021.716114

**Published:** 2021-08-12

**Authors:** Dengwang Liu, Jian Zhan, Zinan Luo, Ningbo Zeng, Wei Zhang, Hao Zhang, Lin Li

**Affiliations:** ^1^College of Agriculture, Hunan Agricultural University, Changsha, China; ^2^Hunan Peanut Engineering and Technology Research Center, Hunan Agricultural University, Changsha, China; ^3^National Peanut Engineering and Technology Research Center, Hunan Agricultural University, Changsha, China; ^4^College of Plant Protection, Hunan Agricultural University, Changsha, China

**Keywords:** data-independent acquisition, malate metabolism, data-dependent acquisition, differentially accumulated proteins, protein-protein interaction, anaerobic respiration enzymes

## Abstract

Peanut is an important oil and economic crop in China. The rainy season (April–June) in the downstream Yangtze River in China always leads to waterlogging, which seriously affects plant growth and development. Therefore, understanding the metabolic mechanisms under waterlogging stress is important for future waterlogging tolerance breeding in peanut. In this study, waterlogging treatment was carried out in two different peanut cultivars [Zhonghua 4 (ZH4) and Xianghua08 (XH08)] with different waterlogging tolerance. The data-independent acquisition (DIA) technique was used to quantitatively identify the differentially accumulated proteins (DAPs) between two different cultivars. Meanwhile, the functions of DAPs were predicted, and the interactions between the hub DAPs were analyzed. As a result, a total of 6,441 DAPs were identified in ZH4 and its control, of which 49 and 88 DAPs were upregulated and downregulated under waterlogging stress, respectively, while in XH08, a total of 6,285 DAPs were identified, including 123 upregulated and 114 downregulated proteins, respectively. The hub DAPs unique to the waterlogging-tolerant cultivar XH08 were related to malate metabolism and synthesis, and the utilization of the glyoxylic acid cycle, such as L-lactate dehydrogenase, NAD^+^-dependent malic enzyme, aspartate aminotransferase, and glutamate dehydrogenase. In agreement with the DIA results, the alcohol dehydrogenase and malate dehydrogenase activities in XH08 were more active than ZH4 under waterlogging stress, and lactate dehydrogenase activity in XH08 was prolonged, suggesting that XH08 could better tolerate waterlogging stress by using various carbon sources to obtain energy, such as enhancing the activity of anaerobic respiration enzymes, catalyzing malate metabolism and the glyoxylic acid cycle, and thus alleviating the accumulation of toxic substances. This study provides insight into the mechanisms in response to waterlogging stress in peanuts and lays a foundation for future molecular breeding targeting in the improvement of peanut waterlogging tolerance, especially in rainy area, and will enhance the sustainable development in the entire peanut industry.

## Introduction

Excessive water in the soil will reduce the rate of gas exchange between the soil and the atmosphere, thus affecting plant growth and development (Andrade et al., 2018; Garcia et al., 2020). Under waterlogging conditions, the oxygen level continues to decrease, but CO_2_, H_2_S, CH_4_, and ethylene continue to accumulate (Kögel-Knabner et al., 2010), which seriously breaks the gas balance and causes a hypoxic or even anoxic environment, reducing the soil reduction potential and affecting the conversion of mineral elements (Ghashghaie et al., 2015). For example, an anoxic environment inhibits aerobic nitrification and nitrifying bacterial activity, resulting in the reduction of nitrate to N_2_ and nutrient loss (Walker et al., 2018). In addition, sulfate is easily reduced to H_2_S under waterlogging condition, reducing the absorption of sulfur by plants and causing toxicity to plants (Pezeshki and DeLaune, 2012). Waterlogging also reduces oxidized compounds, such as Fe^2+^ and Mn^4+^, raising up the concentration level of Fe and Mn beyond the nutritional requirements in plants (Khabaz-Saberi et al., 2006; Khabaz-Saberi and Rengel, 2010).

The plant morphology will also be adversely affected under waterlogging stress. The anoxic environment usually decreases leaf resistivity, increases cell electrolyte leakage, and destroys the filtering function of cell membrane. In addition, the chlorophyll level and its synthetic rate were reduced, leading to leaf yellowing and wilting, accelerating the leaf shedding rate, promoting leaf senescence, and hindering the formation of new leaves (Zheng et al., 2009). Waterlogging stress also inhibits root growth, reduces root hair, and decays root tips (Xuewen et al., 2014). In order to adapt to this condition, adventitious roots were formed in large quantities and became the main components of the root system under waterlogging stress, thus expanding the absorption area of plants, improving the absorption and transportation of oxygen, and therefore giving the cells a higher mitotic ability and physiological activity (Zou et al., 2010). Another adaptation in plants under waterlogging stress is to facilitate the accumulation of ethylene concentration (Kim et al., 2018; Zhao et al., 2018), which leads to an increase in cellulase activity, thus resulting in the separation and collapse of root tip cortical cells and the formation of aerenchyma (a gas channel composed of specific cells that can transport oxygen to the root system and alleviate the pressure of oxygen deficiency) (Wang et al., 2016). This adaptation strategy helps maintain the normal physiological metabolism of root cells.

Waterlogging also affects metabolic processes, such as photosynthesis in plants. Under waterlogging stress, the photosynthetic rate in waterlogging-tolerant plants did not change too much, while it dropped rapidly in waterlogging-sensitive plants (Zeng et al., 2020). In addition, waterlogging stress leads to oxygen shortage, which will trigger anaerobic respiration to provide cell energy (da Silva and do Amarante, 2020). Anaerobic respiration usually includes two fermentation pathways: lactic acid and ethanol fermentation. Thus, the enzymes involved in these fermentation pathways, such as lactate dehydrogenase (LDH) and alcohol dehydrogenase (ADH), are changed accordingly as indicators. For example, ADH and LDH will be activated to provide plants with ATP to improve the ability of plants to cope with waterlogging stress (Dennis et al., 2000; Xu et al., 2016).

Most previous studies mainly focused on the effects of waterlogging stress on plant morphology, physiology, dry matter accumulation and distribution, crop yield, and quality characteristics. Few studies have been conducted to unveil the molecular mechanisms under waterlogging stress in peanut. We therefore undertook a quantitative proteomics study to provide a comprehensive understanding of peanut roots in response to waterlogging stress. A number of techniques and strategies can be used for quantitative proteomics study, among which isotopic labeling (e.g., iTRAQ, SILAC, or TMT) and label-free methods are the two techniques with highest popularity (Jylhä et al., 2018). The isotopic labeling strategy relies on binding-specific isobaric labels to peptides for relative quantification of proteins, but it limits the number of different experimental groups (usually only 4–8 samples; Ross et al., 2004; Choe et al., 2012). Data-dependent acquisition (DDA) is often used in iTRAQ, which cannot be applied to large-scale trials due to high cost, setup complexity, and incomplete selection of peptides (Jylhä et al., 2018). However, the sequential windowed acquisition of all theoretical fragment ion mass spectra (SWATH) is a data-independent acquisition (DIA) technique that can overcome this difficulty. SWATH is label-free and not limited to the number of experimental groups, allowing for flexible comparisons and potential savings (Gillet et al., 2012). In addition, SWATH obtains ion spectra containing all the fragments from all the precursor ions within the predefined mass-to-charge (m/z) range (Gillet et al., 2012). The independent datasets obtained from DIA can be re-examined if the library used in the downstream analyses is updated (Jylhä et al., 2018).

In the current study, DIA technique is used to quantitatively analyze the differentially accumulated proteins (DAPs) between two peanut cultivars with different waterlogging tolerance: Xianghua08 (XH08, waterlogging-tolerant) and Zhonghua 4 (ZH4, waterlogging-sensitive). In addition, the effects of waterlogging stress on the relative respiratory enzymes and root morphology were observed and compared between two cultivars. This study provided a theoretical basis for future waterlogging tolerance breeding in peanut and will be of great significance for the sustainable development of the peanut industry.

## Materials and Methods

### Plant Materials and Waterlogging Treatment

The tested peanut cultivars, including waterlogging-sensitive cultivar ZH4 and the waterlogging-tolerant cultivar XH08, were bred by the Arid Land Crops Research Institute of Hunan Agricultural University (Changsha, Hunan). Stainless steel brackets filled with clean fine quartz sand were placed in the PVC basins with a diameter of 20 cm and height of 3 cm, and one seed was sown in each basin in 3 cm depth, with each cultivar replicating three times. When the seedlings germinated to the stage of four leaves and one heart, the flooding treatment was carried out to maintain a flooding depth of 1 cm for different duration time (3, 6, 9, 12, and 15 days). The control group was irrigated normally. The light and dark conditions were alternated with 14-h daylight time and 10-h dark conditions. The temperature was set to 25°C (daylight) and 20°C (night), respectively.

### Plant Root Microscopy and Determination of Enzymatic Activities

After waterlogging treatment, paraffin sections of main roots of two cultivars and their controls were made, and the microstructure of the root tissue was observed. The taproots of peanut cultivars (ZH4 and XH08) and their controls were collected for the determination of LDH, ADH, and malate dehydrogenase (MDH) activities as follows: 0.1 g taproot samples of waterlogging-treated cultivars and their control samples were weighed after 12 days and 15 days of waterlogging treatment with each group replicating three times. The supernatants were homogenized on an ice bath with 1 ml of 0.1 M PBS buffer, followed by centrifugation under 4°C at 15,000 rpm for 20 min. The wavelength of the ultraviolet spectrophotometer was set to 340 nm (zero adjustment of ultrapure water). The activities of LDH, ADH, and MDH in peanut taproots were determined using the LDH assay kit (A020-1), the ADH assay kit (A083-2-1), and the MDH assay kit (A021-2-1; Nanjing Jiancheng Bioengineering Research Institute) following the manufacturer’s instructions. Exploring changes in enzymatic activities aimed to determine the time point at which the root samples would be collected for the following quantitative proteomics analyses. The collected root samples were immediately frozen in −80°C for further use.

### Protein Extraction and Digestion

Proteomic sequencing was performed by the Beijing Genome Institute (Shenzhen, China). The collected peanut roots were first ground using 5-mm magnetic beads, and proteins were extracted using lysis buffer 3 (10 mm Tris–HCl, pH 8.0, 100 mm NaCl, 1 mm EDTA, 0.5 mm EGTA, 0.1% sodium deoxycholate, and 0.5% sodium N-lauroyl sarcosine), with PMSF (protease inhibitor), EDTA, and dithiothreitol (DTT) added to a final concentration of 1 mm, 2 mm, and 10 mm, respectively. The mixture was ground using a tissue grinder for 2 min (power = 50 Hz, time = 120 s), followed by centrifugation at 25,000 g under 4°C for 20 min. After the removal of the supernatant, 10 mm DTT was added, and the mixture was heated at 56°C for 1 h. Iodoacetamide (55 mm) was then added to the mixture and incubated in the dark for 45 min. The protein was precipitated with four volumes of cold acetone for 2 h at −20°C. This step was repeated 2–3 times until the supernatant turned to be transparent. After centrifugation at 25,000 g under 4°C for 20 min, the precipitation was redissolved in lysis buffer 3 and ground using tissue grinder for 2 min (power = 50 Hz, time = 120 s), followed by centrifugation at 25,000 g under 4°C for 20 min. The supernatant was retained for protein quantification using the Bradford method (Kruger, 2009), and the extracted proteins were separated using SDS-PAGE. After electrophoresis, the solution was stained in Coomassie Brilliant Blue for 2 h, followed by decolorization using the decolorizing solution (40% ethanol and 10% acetic acid) for 3–5 times.

Each protein sample (~100 μg protein) was digested twice using trypsin at a 1:40 trypsin-to-protein ratio at 37°C for 4 h, followed by another round at 37°C for 8 h. After trypsin digestion, the peptides were desalted using a Strata-X column (Phenomenex, Los Angeles, United States) and vacuum dried.

### HPLC Fractionation and LC MS/MS Under Both DDA and DIA Modes

The peptide samples were fractionated using high-pH reversed-phase high-performance liquid chromatography by using the Gemini C18 column (5 μm, 4.6 mm × 250 mm). Briefly, peptides were first separated with a gradient of 5 to 95% acetonitrile (pH 9.8) over 60 min. Next, they were combined into 10 fractions and freeze-dried by vacuum centrifugation. The dried peptide samples were redissolved with mobile phase A (2% ACN, 0.1% FA) and centrifuged at 20,000 g for 10 min, and the supernatant was retained for MS analysis. LC–MS/MS was carried out in the ultimate 3,000 UHPLC (Thermo scientific, United States). The sample was first enriched and desalted using a trap column and then connected to a self-contained C18 column (150 μm inner diameter, 1.8 μm column material particle size, 25 cm column length) in series. Separation was carried out at a flow rate of 500 nl/min through the following concentration gradient: 0–5 min, 5% mobile phase B (98% ACN, 0.1%). Mobile phase B increased linearly from 5 to 35% from 5 to 160 min, increased from 35 to 80% over 160–170 min, and then increased to 80% over 170–175 min, and finally decreased to 5% over 176–180 min. Afterward, the peptides were injected into a nanoESI ion source followed by a tandem mass spectrometer for both DDA and DIA analyses.

#### Data-Dependent Acquisition Mass Spectrometry

The separated peptides were ionized by the nanoESI source and injected into a Q-Exactive HF tandem mass spectrometer (Thermo Fisher Scientific, San Jose, CA) under DDA mode. Briefly, intact peptides were detected in the Orbitrap at a resolution of 60,000 with a MS range of 350–1,500 m/z for full scan. The 20 most intense precursor ions per survey scan were selected for higher-energy collisional dissociation (HCD) fragmentation, and the resulting fragments were analyzed with the Orbitrap at a resolution of 15,000 with a fixed mass of 100 m/z. The MS was operated between MS scan and MS/MS scan with the dynamic exclusion time of 30s. The automatic gain control (AGC) was set as 3E6 for level 1 and 1E5 for level 2.

#### Data-Independent Acquisition Mass Spectrometry

Another batch of separated peptides was injected into the Q-Exactive HF tandem mass spectrometer (Thermo Fisher Scientific, San Jose, CA) under DIA mode. Briefly, intact peptides were detected in the Orbitrap at a resolution of 120,000 with a MS range of 350–1,500 m/z for full scan, followed by the division of 40 windows for fragmentation and signal acquisition. The fragmentation mode was HCD, and ion fragments were detected with Orbitrap. The dynamic exclusion time was set to 30 s. The AGC was set as follows: 3E6 for level 1 and 1E5 for level 2.

### Database Search

The Andromeda engine implemented in MaxQuant was used to process DDA data obtained from the machine.^1^ Spectronaut was then used to construct a spectrum library and complete the data deconvolution. The mProphet algorithm was used to complete the data analysis and quality control. Based on the UniProt protein databases, GO, COG/KOG and KEGG pathway annotations were conducted. MSstats software was used to preprocess the data and make comparisons among different group sets. The threshold of significance level for DAPs was set at fold change (FC) ≥ 2 and *p* < 0.05. Finally, enrichment analysis and subcellular localization analysis were performed for DAPs, and protein-protein interaction (PPI) analysis was conducted using STRING database for the functional classification of DAPs.

### Data Analysis

SPSS software (v19.0, IBM Corp., Armonk, NY) was used to analyze different respiratory enzymatic activities between two cultivars and their controls. Origin 2019 software was used to draw bar charts and dot charts. The volcano diagram of DAPs between two peanut cultivars and their controls (| log2FC | ≥ 1 Magi, *p* < 0.05) was depicted using EXCEL, and the KEGG pathway enrichment bubble diagram was generated by R (R Development Core Team, 2010). The key nodes of DAPs with a confidence score over 500 in the PPI interaction network were selected, and the MCC algorithm implemented in the cytoHubba was used to analyze and identify the hub DAPs using the Cytoscape software (Paul Shannon et al., 1971). The top 100 DAPs with highest confidence levels were used to draw the PPI interaction network diagram.

## Results

### Effects of Waterlogging Stress on the Morphology of Peanut Taproots

**Figure 1:**
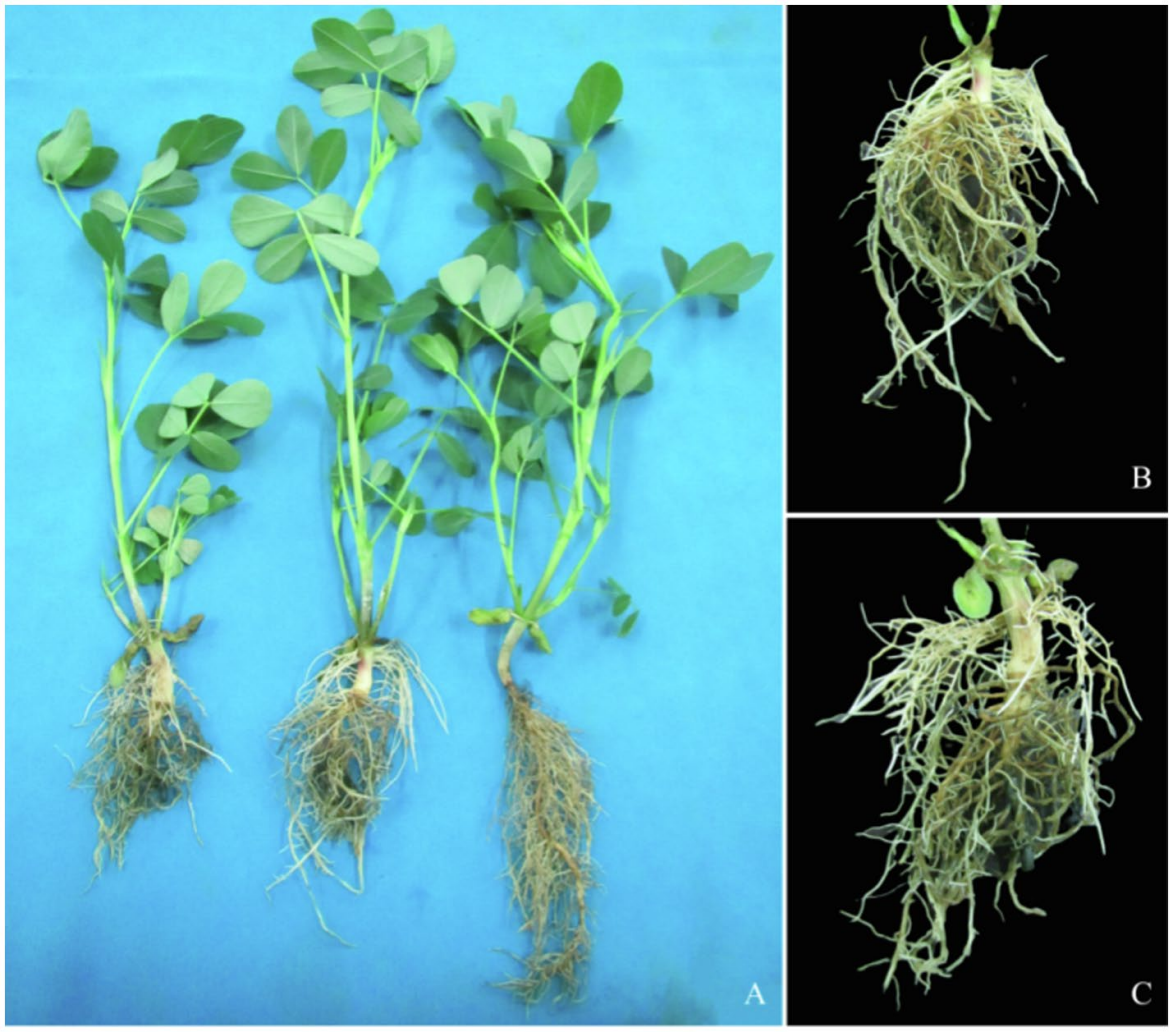
Morphological differences between two treated peanut cultivars and their controls. **(A)** The peanut taproots of treated and the control groups after 22 days of waterlogging treatment. Left two samples were treated ones, and the right one was control sample; **(B)** the peanut hypocotyl produced adventitious roots in Zhonghua 4 (ZH4) after 22 days waterlogging treatment; and **(C)** the peanut epicotyl produced adventitious roots in Xianghua08 (XH08) after 22 days of waterlogging treatment.

Plant roots are responsible for nutrient and water transportation. When external conditions change, plants adapt to the environment by changing root morphological structure. As shown in Figure 1A, after 22 days of waterlogging treatment, the roots of two cultivars became dark brown and rotten, and the growth and development of main roots in controls were better than the treated cultivars. The hypocotyl (Figure 1B) of ZH4 generated obvious adventitious roots, while the epicotyl (Figure 1C) of XH08 generated obvious adventitious roots.

**Figure 2:**
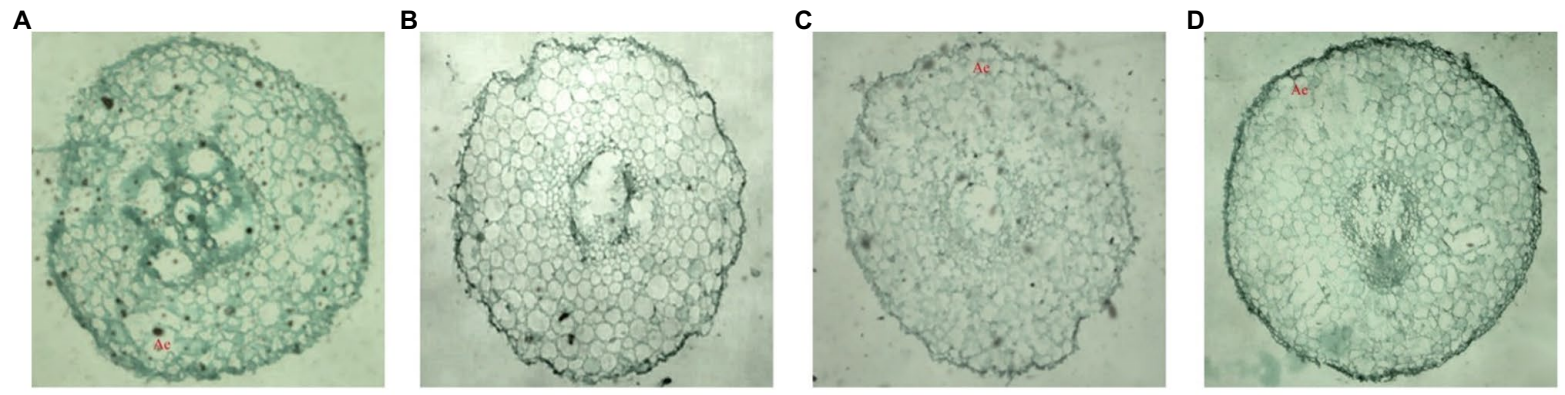
The root tissue micrographs (aerenchyma) of the main roots in two peanut cultivars after waterlogging for 6 days and 9 days. **(A)** Root microscopic map of ZH4 after 6 days of waterlogging treatment; **(B)** root tissue microscopic map of XH08 after 6 days of waterlogging treatment; **(C)** root microscopic map of ZH4 after 9 days of waterlogging treatment; and **(D)** root microscopic map of XH08 after 9 days pf waterlogging treatment.

After 3 days of waterlogging treatment, there was no significant difference in the root growth between ZH4 and XH08, and no ventilatory tissue was found. However, after waterlogging for 6 days, most of the cortical parenchyma cells in ZH4 taproots were dissociated to form ventilatory tissue, while the cortical parenchyma cells in XH08 enlarged and deformed without dissociation, and no obvious ventilatory tissues were formed (Figures 2A,B). After 9 days of waterlogging, the xylem in both cultivars showed irregular distribution and a large number of parenchyma cells were damaged. However, the amount of XH08 ventilatory tissue increased without the damage of the cell wall (Figures 2C,D).

### Functional Analysis of DAPs in Different Peanut Cultivars Under Waterlogging Stress

#### The DAPs Induced by Waterlogging Stress

**Figure 3:**
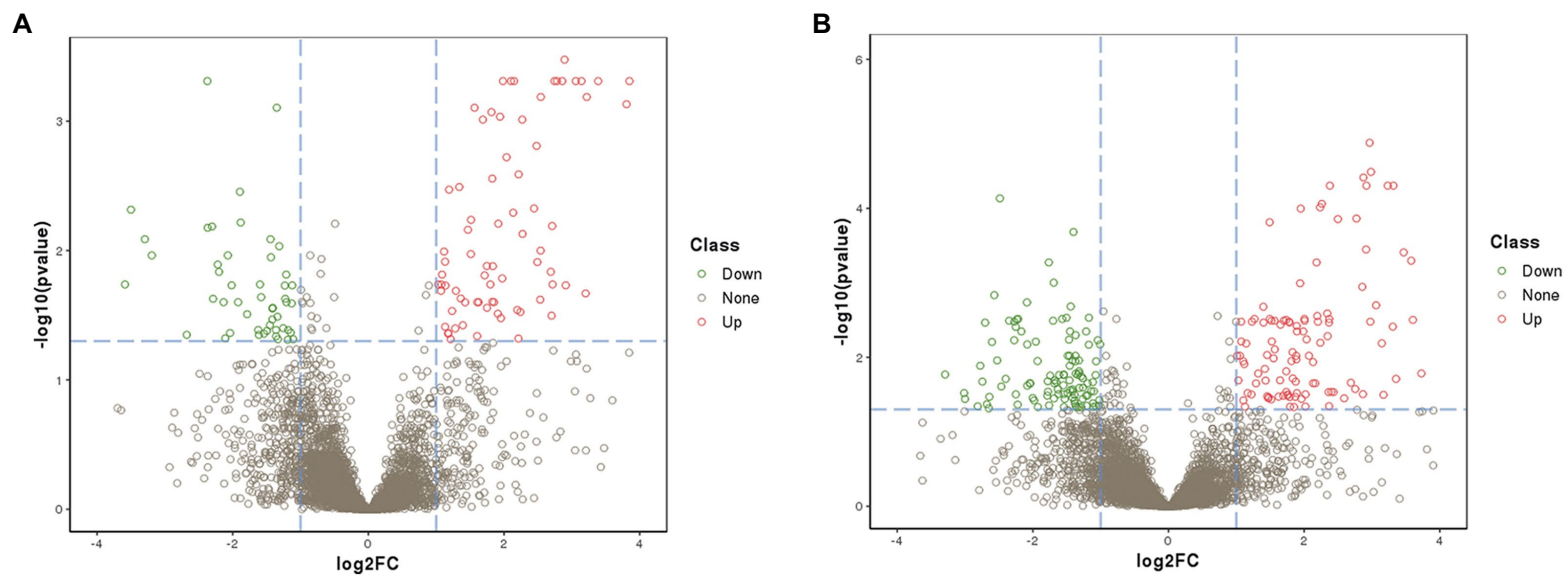
Volcano map of differentially accumulated proteins (DAPs) in two different peanut cultivars after 12 days of waterlogging treatment. **(A)** Volcano map of DAPs between ZH4 and its control group after 12 days of waterlogging treatment. **(B)** Volcano map of DAPs between XH08 and its controls after 12 days of waterlogging treatment.

**Figure 4:**
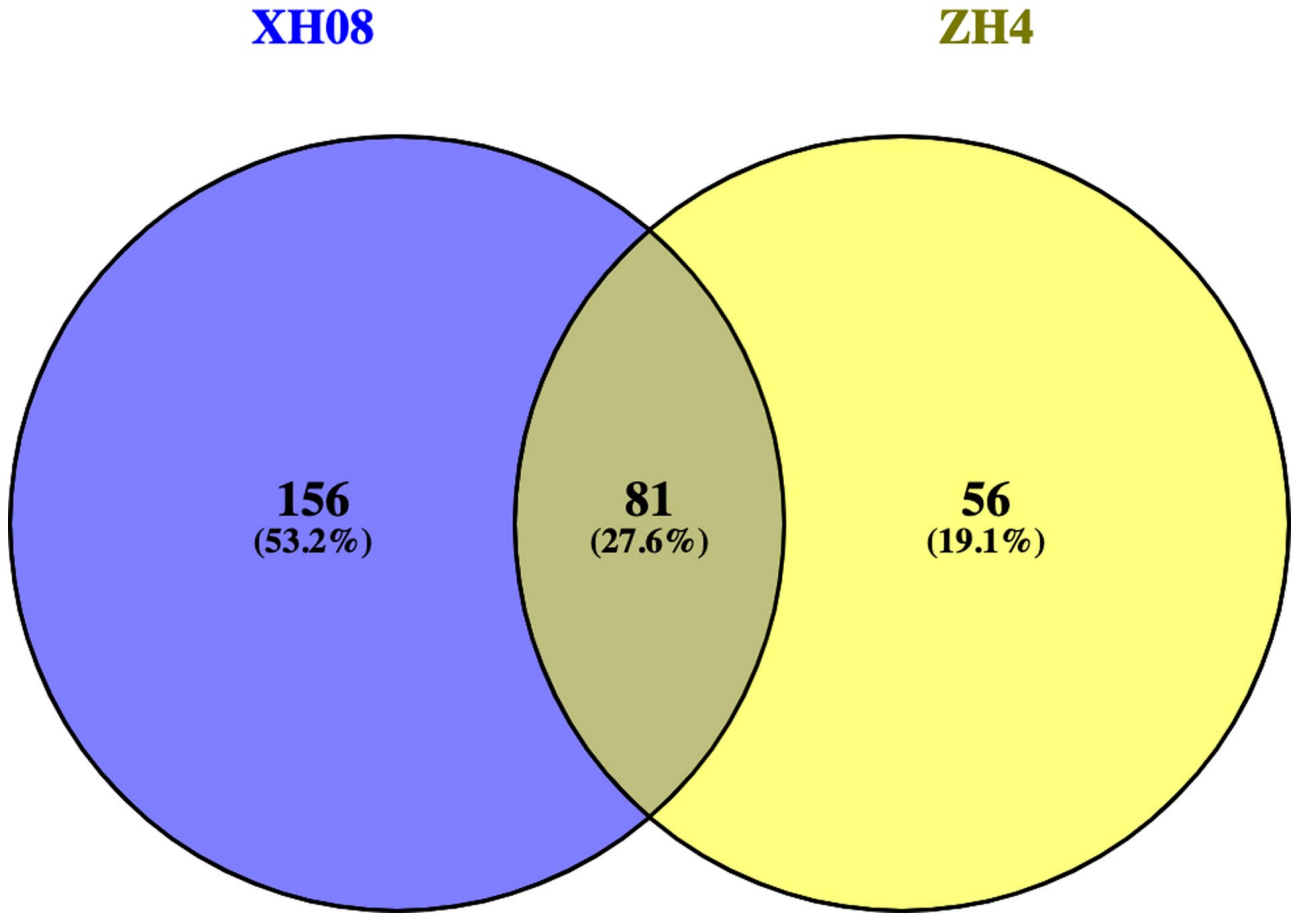
The Venn diagram indicating a comparison of DAPs between XH08 and ZH4.

Quantitative proteomic analysis of two peanut cultivars with different waterlogging tolerance was carried out by the DIA technique. The analysis of significant DAPs between the treatment and control samples is shown in a volcanic map (Figure 3). As shown in Figure 3A, a total of 6,441 DAPs were identified between the treated ZH4 and its control group, of which 49 DAPs were significantly upregulated and 88 DAPs were significantly downregulated (| log2FC | ≥ 1, *p* < 0.05). As shown in Figure 3B, a total of 6,285 DAPs were identified between the treated XH08 and its controls, of which 123 and 114 DAPs were significantly upregulated and downregulated, respectively (| log2FC | ≥ 1, *p <* 0.05). Among these DAPs, a total of 156 and 56 specifically existed in XH08 and ZH4, respectively (Figure 4), and 81 DAPs shared between two cultivars. In brief, the number of significant DAPs in the waterlogging-tolerant cultivar XH08 was higher than the sensitive cultivar ZH4.

#### KEGG Pathway Enrichment Analysis

**Figure 5:**
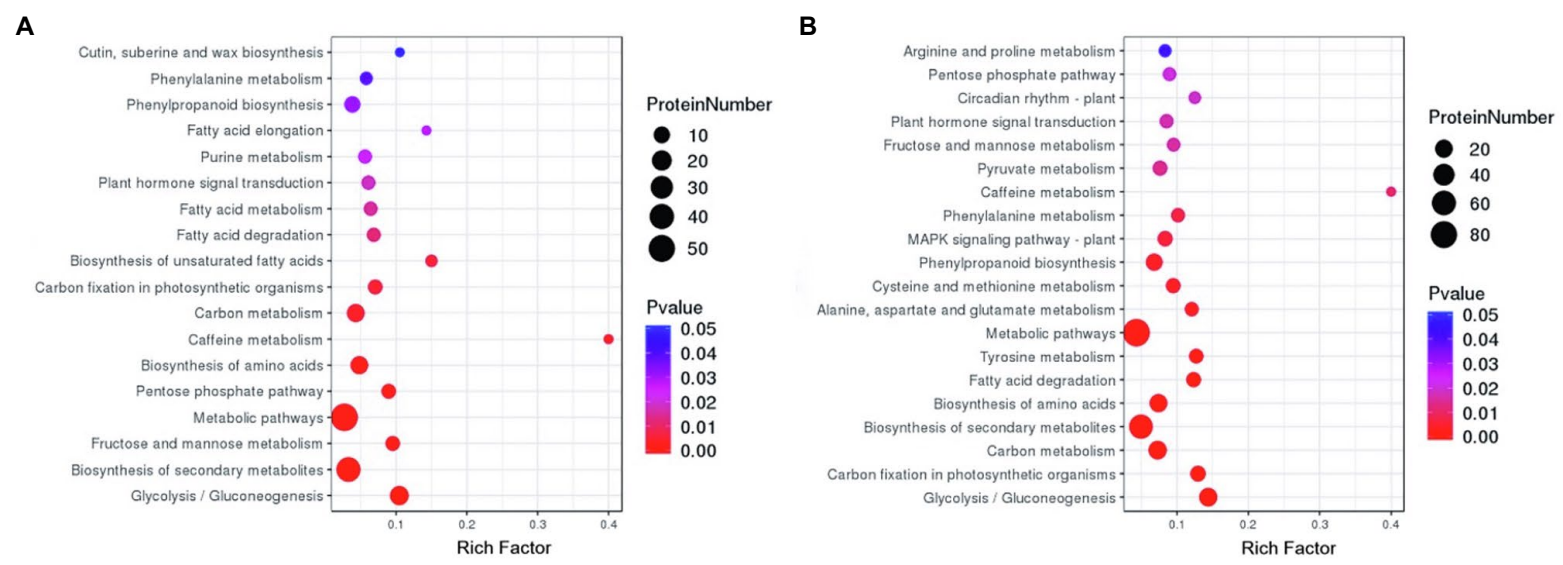
The KEGG signaling pathways of DAPs in two different peanut cultivars were enriched by a bubble map after 12 days of waterlogging treatment. **(A)** Bubble map of the significantly enriched KEGG pathways of DAPs in ZH4 and its controls after 12 days of waterlogging treatment. **(B)** Bubble map of DAPs in KEGG pathways between XH08 and its control samples after 12 days of waterlogging treatment.

**Figure 6:**
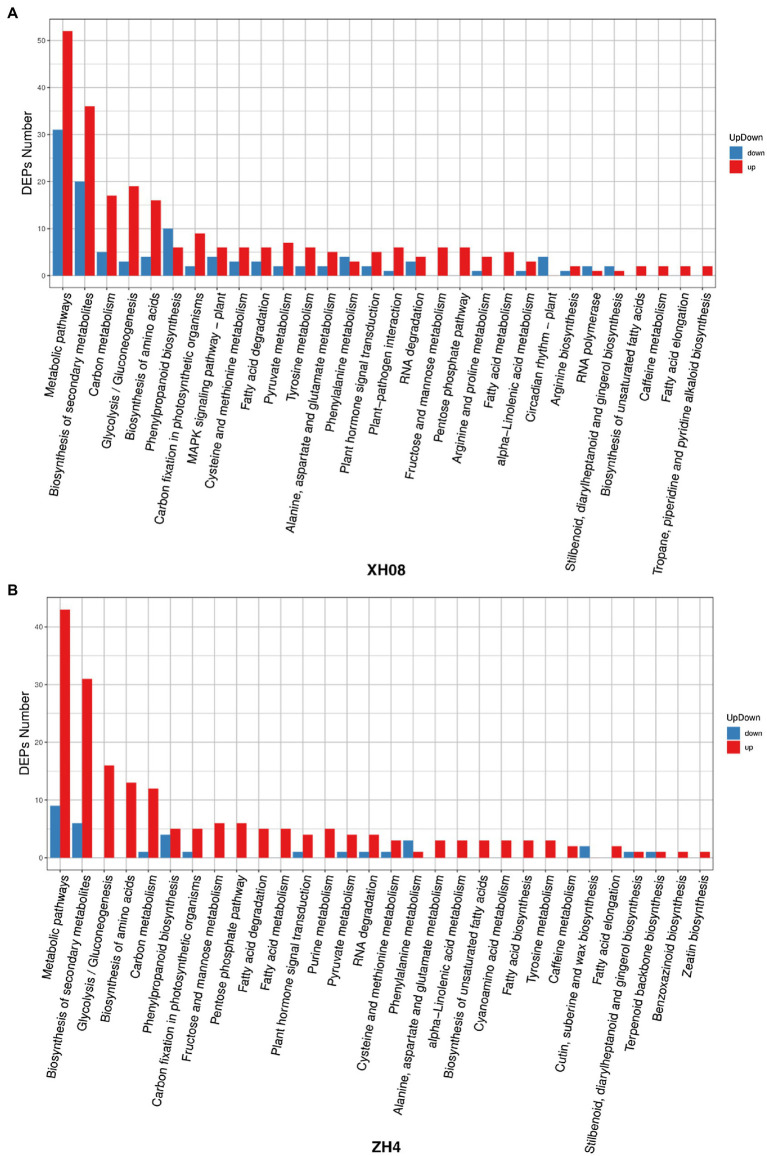
The up- and downregulated DAPs in two peanut cultivars and their controls. **(A)** Up- and downregulated KEGG pathways of DAPs in ZH4 and its controls; **(B)** up- and downregulated KEGG pathways of DAPs in XH08 and its controls.

The DAPs related to waterlogging in two peanut cultivars were enriched by KEGG pathway enrichment analysis (Figure 5). According to the significance of waterlogging-related DAP enrichment in the signaling pathway, the waterlogging-related DAPs in ZH4 were mainly enriched in the following six metabolic pathways (the number of DAPs enriched in each signaling pathway is in parentheses; Figure 5A): glycolysis/gluconeogenesis (16), secondary metabolites (37), fructose and mannose metabolism (6), metabolic pathways (52), pentose phosphate pathway (6), and amino acid biosynthesis (13). The waterlogging-related DAPs in XH08 were mainly enriched in the following six metabolic pathways (Figure 5B): glycolysis/gluconeogenesis (22), carbon fixation in photosynthetic organisms (11), carbon metabolism (22), secondary metabolites (56), amino acid biosynthesis (20), and fatty acid degradation (9). In brief, the main enriched signaling pathways shared between ZH4 and XH08 included metabolic pathways, secondary metabolites, glycolysis/gluconeogenesis, carbon metabolism, amino acid biosynthesis, and phenylpropanoid biosynthesis. The relative up- and downregulated KEGG pathways of the DAPs in both XH08 and ZH4 were indicated in Figure 6, indicating that although the trends in upregulated KEGG pathways were similar between two cultivars, more DAPs were downregulated across KEGG pathways in XH08 than ZH4.

#### Hub DAP PPI Interaction Network Analysis

**Figure 7:**
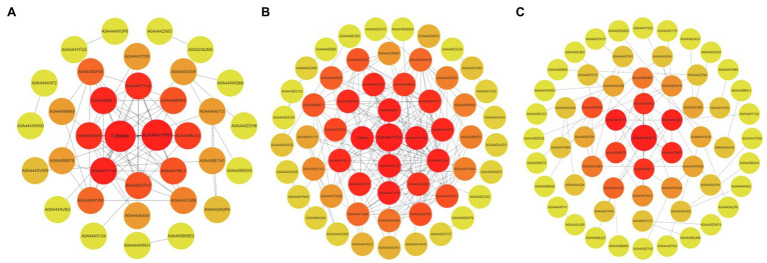
The protein-protein interaction (PPI) network interaction map of the differentially accumulated proteins (DAPs) in two peanut cultivars after 12 days of waterlogging treatment. **(A)** PPI interaction network map of hub DAPs between ZH4 and its control group after 12 days of waterlogging treatment; **(B)** PPI interaction network map of hub DAPs in XH08 and its control group after 12 days of waterlogging treatment; **(C)** PPI interaction network map of hub DAPs between XH08 and ZH4 subjected to waterlogging treatment.

The key nodes with the confidence score over 500 in the PPI interaction network were selected, and the MCC algorithm of the Cytoscape software cytoHubba was used to find the hub DAPs (Figure 6). The number of key DAPs (i.e., PPI interaction network key nodes) in XH08 and the number of interactions between key DAPs were higher than ZH4 (Figure 7). The hub DAPs related to waterlogging in ZH4 mainly included T2B9M0, A0A444YP93, A0A444YY24, and A0A445B9L1 (Table 1; Figure 7A). In addition, A0A444YYU4, A0A444XSI4, A0A445BU22, A0A445CFH7, A0A444Y8U1, and A0A445B0R4 also played important roles in response to waterlogging stress, while the only different hub DAP in XH08 from ZH4 was A0A444Y0J8 (Table 1; Figure 7B). Compared to ZH4, the DAPs, such as A0A444WVT0, A0A445AX60, A0A444ZM03, A0A445B4C1, A0A444X6X2, and A0A445ARZ8, played a key role in the physiological process of XH08 under waterlogging stress (Table 1; Figure 7C). Under waterlogging stress, the common hub DAPs between XH08 and ZH4 mainly included the enzymes related to glycolysis pathway, such as enolase, fructose-bisphosphate aldolase, glyceraldehyde-3-phosphate dehydrogenase, ATP-dependent 6-phosphofructokinase, and pyruvate kinase, while the unique hub DAPs in XH08 mainly included malate metabolism, such as L-lactate dehydrogenase, NAD^+^-dependent malic enzyme, aspartate aminotransferase, and glutamate dehydrogenase as well as the enzymes related to glyoxylic acid cycle. Details of hub DAPs and the corresponding functional predictions between two peanut cultivars are shown in Table 1.

**Table 1:** The hub DAPs (confidence score ≥ 500) in the interaction network between XH08, ZH4, and their control groups.

Protein ID	SWISS-PROT description (function)	Log2FC(up/down)	Value of *p*
*ZH4*(sensitive cultivar)hub DAPs			
T2B9M0	Fructose-bisphosphate aldolase, cytoplasmic isozyme	1.1374/Up	0.00024
A0A444YP93	Fructose-bisphosphate aldolase 8, cytosolic	1.1311/Up	0.00012
A0A444YY24	Enolase	2.1478/Up	5.86E-07
A0A445B9L1	Glyceraldehyde-3-phosphate dehydrogenase GAPC2, cytosolic	2.0370/Up	8.28E-06
A0A444YYU4	Pyruvate kinase, cytosolic isozyme	1.4266/Up	0.00042
A0A444XSI4	Pyruvate kinase 1, cytosolic	2.2689/Up	3.93E-06
A0A445BU22	ATP-dependent 6-phosphofructokinase 3	1.8147/Up	2.91E-06
A0A445CFH7	ATP-dependent 6-phosphofructokinase 3	2.7801/Up	9.14E-07
A0A444Y8U1	ATP-dependent 6-phosphofructokinase 3	3.2192/Up	1.52E-06
A0A445B0R4	Pyrophosphate-fructose 6-phosphate 1-phosphotransferase subunit alpha	1.0303/Up	0.00022
*XH08*(waterlogging-tolerant cultivar) hub DAPs			
A0A444YY24	Enolase	2.3791/Up	7.18E-08
T2B9M0	Fructose-bisphosphate aldolase, cytoplasmic isozyme	1.3983/Up	1.33E-05
A0A444YP93	Fructose-bisphosphate aldolase 8, cytosolic	1.2305/Up	3.62E-05
A0A444Y0J8	Phosphoglycerate kinase 3, cytosolic	1.0122/Up	0.00017
A0A445B9L1	Glyceraldehyde-3-phosphate dehydrogenase GAPC2, cytosolic	2.1833/Up	2.51E-06
A0A444YYU4	Pyruvate kinase, cytosolic isozyme	1.9405/Up	5.32E-06
A0A444XSI4	Pyruvate kinase 1, cytosolic	2.8732/Up	3.68E-08
A0A445ASF0	L-lactate dehydrogenase A	1.1019/Up	0.00022
A0A445CFH7	ATP-dependent 6-phosphofructokinase 3	1.8693/Up	0.00121
A0A444Y8U1	ATP-dependent 6-phosphofructokinase 3	2.1321/Up	0.00061
*ZH4* vs. *XH08* hub DAPs			
A0A445ASF0	L-lactate dehydrogenase A	1.1019/Up	0.00022
A0A444WVT0	Phosphoenolpyruvate carboxykinase	−1.6541/Down	0.00057
A0A445AX60	Pyruvate, phosphate dikinase, and chloroplastic	3.1700/Up	0.00108
A0A444ZM03	NAD^+^-dependent malic enzyme 59 kDa, mitochondrial	1.5232/Up	9.15E-05
A0A445B4C1	Aspartate aminotransferase 1	1.4908/Up	5.38E-07
A0A444X6X2	Aspartate aminotransferase 1	1.7860/Up	3.93E-05
A0A445ARZ8	Glutamate dehydrogenase A	−1.0087/Down	0.000108

### Effects of Waterlogging Stress on Enzymatic Activities in Two Peanut Cultivars

**Figure 8:**
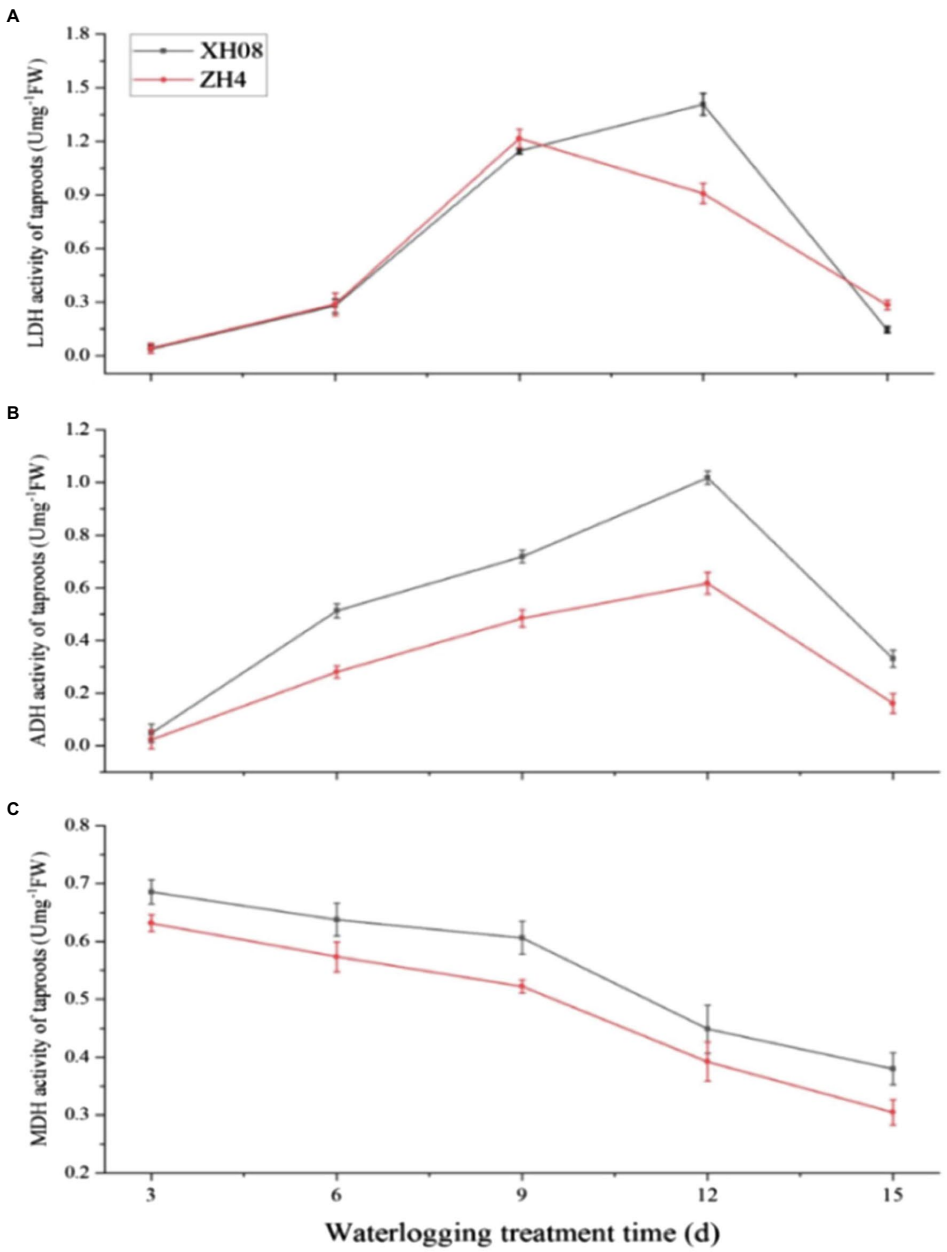
The activities of LDH, alcohol dehydrogenase (ADH), and malate dehydrogenase (MDH) in two peanut cultivars treated with waterlogging for 12 and 15 days. **(A)** The activities of the LDH enzyme in XH08 and ZH4 after waterlogging treatment for 3, 6, 9, 12, and 15 days; **(B)** the activities of the ADH enzyme in XH08 and ZH4 after waterlogging treatment for 3, 6, 9, 12, and 15 days; **(C)** the activities of the MDH enzyme in XH08 and ZH4 after waterlogging treatment for 3, 6, 9, 12, and 15 days.

The activities of LDH, ADH, and MDH in the main roots of two peanut cultivars were measured after waterlogging treatment for 3, 6, 9, 12, and 15 days, respectively, and the change of enzymatic activities across waterlogging treatment was analyzed (Tables 2 and 3; Figure 8). The LDH activity of the main roots of ZH4 and XH08 increased slightly slow from 3 to 6 days. However, a sharp increase was observed after treatment for 6 days and reached to a peak on the ninth day in ZH4, while the LDH activity in XH08 continued to increase until reaching a peak on the twelfth day. The LDH activity of in two cultivars showed a downward trend from 12 to 15 days, and a sharper decrease was observed in XH08 than ZH4. However, the ADH activity in both cultivars continuously increased and reached a peak until twelfth day, with the decrease in XH08 being greater than ZH4. Unlike the above two enzymes, the MDH activity in ZH4 and XH08 continuously dropped from 3 to 15 days.

**Table 2:** Enzymatic activities of LDH, ADH, and MDH in XH08 and ZH4 treated with waterlogging for 3, 6, 9, 12, and 15 days (U mg^−1^ FW).

Enzyme	Cultivar	Waterlogging treatment time (*d*)				
		3d	6d	9d	12d	15d
LDH	XH08	0.037 ± 0.024	0.278 ± 0.037	1.145 ± 0.015	1.406 ± 0.061	0.145 ± 0.018
	ZH4	0.041 ± 0.028	0.287 ± 0.064	1.216 ± 0.053	0.907 ± 0.056	0.283 ± 0.027
ADH	XH08	0.047 ± 0.035	0.514 ± 0.027	0.719 ± 0.023	1.018 ± 0.025	0.330 ± 0.033
	ZH4	0.023 ± 0.035	0.281 ± 0.024	0.484 ± 0.032	0.617 ± 0.042	0.159 ± 0.037
MDH	XH08	0.685 ± 0.021	0.637 ± 0.028	0.606 ± 0.028	0.448 ± 0.041	0.381 ± 0.027
	ZH4	0.631 ± 0.014	0.573 ± 0.026	0.522 ± 0.011	0.392 ± 0.034	0.304 ± 0.022

**Table 3:** ANOVA analysis of the enzymatic activities (U mg^−1^ FW) of ADH and LDH in XH08 and ZH4 treated with and without waterlogging after 12 days.

Enzyme	Cultivar	Enzyme activity (U mg^−1^ FW)		Significance level
		Treated with waterlogging	Treated without waterlogging (CK)	
LDH	ZH4	0.907 ± 0.056	0.461 ± 0.016	^**^
	XH08	1.406 ± 0.061	0.791 ± 0.026	^***^
ADH	ZH4	0.617 ± 0.042	0.024 ± 0.016	^*^
	XH08	1.018 ± 0.025	0.758 ± 0.024	^***^

* *p* ≤ 0.05;

** *p* ≤ 0.01; and

*** *p* ≤ 0.001.

**Figure 9:**
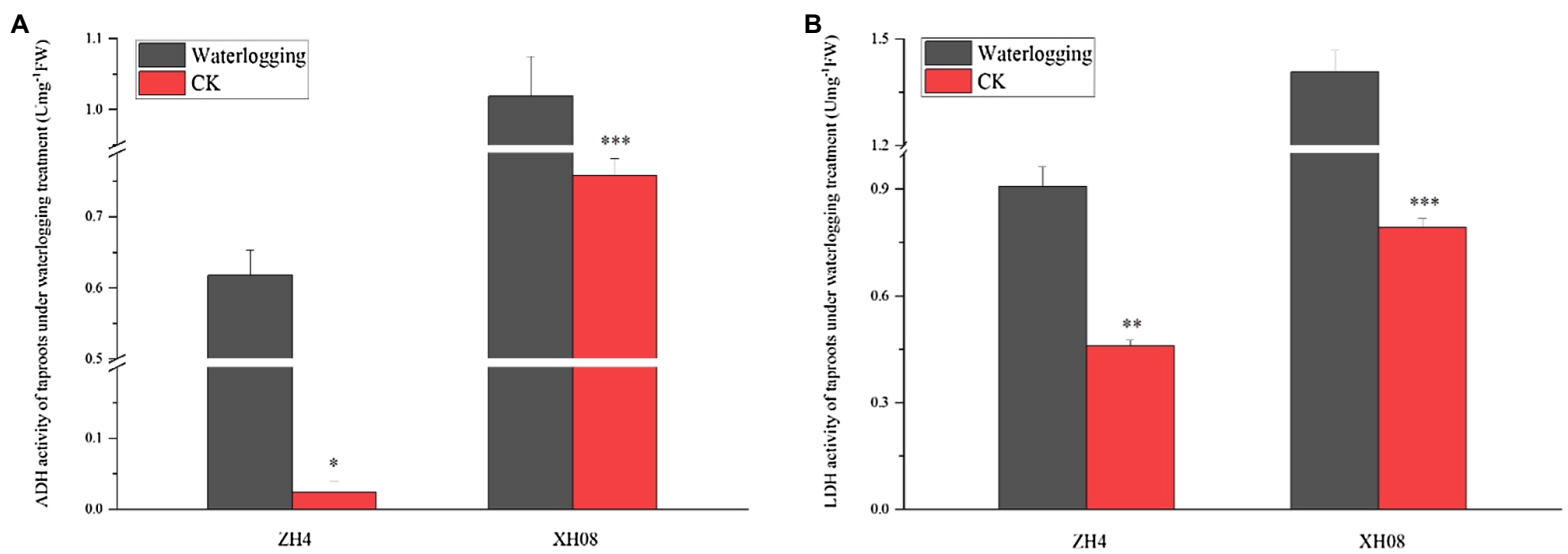
The difference in the activities of ADH and LDH (U mg^−1^ FW) in two different peanut cultivars after 12 days of waterlogging treatment. **(A)** The difference in ADH activity (U mg^−1^ FW) between ZH4 and XH08 after 12 days of waterlogging treatment. **(B)** The difference in LDH activity after 12 days of waterlogging treatment between ZH4 and XH08 (^*^p < 0.05; ^**^p < 0.01; ^***^p < 0.001).

Based on the above results, both ADH and LDH activities reached to a peak at the twelfth day in XH08. At this point of time, the activities of ADH and LDH in both treated cultivars were significantly higher than control groups (Figure 9), with the *p*-value between treated XH08 and its controls in both ADH and LDH activities smaller than 0.001 and the value of *p* between treated ZH4 and its controls in ADH and LDH smaller than 0.05 and 0.01, respectively.

## Discussion

It was found that the production of adventitious roots is an important way for plants to adapt to waterlogging stress. In order to easily obtain oxygen from the environment to maintain normal physiological functions, most of the adventitious roots grow at the base of the stem, on the surface of water or on the surface of soil rich in oxygen after waterlogging stress (Xu et al., 2017). In our study, after 22 days of waterlogging treatment, obvious adventitious roots were grown on both the epicotyl and hypocotyls of main roots in treated peanut cultivars (Figure 1). In previous studies, researchers found that under waterlogging stress, plant root parenchyma cells usually died or dissolved to form lytic aerated tissue, which was convenient to transport oxygen from aboveground parts to roots in the soil to maintain the normal physiological functions (Yamauchi et al., 2018; Peng et al., 2020). In agreement with the above findings, our study also found that the dissociation of parenchyma cells from the cortex in ZH4 began to form aerenchyma slightly earlier than XH08 (Figure 2), and as the waterlogging treatment time increased, a large number of parenchyma cell walls were damaged, and it was difficult to maintain normal morphology. However, the aerenchyma in XH08, a waterlogging-tolerant cultivar, appeared later, resulting in normal cell wall (Figure 2).

The KEGG pathway analysis (Figure 5) showed that waterlogging-related DAPs in two peanut cultivars were both significantly enriched in the glycolysis/gluconeogenesis signaling pathway, and the PPI interaction analysis showed that the common function of hub DAPs in two cultivars was also related to the biosynthesis of glycolysis-related enzymes, such as enolase, fructose-bisphosphate aldolase, glyceraldehyde-3-phosphate dehydrogenase, ATP-dependent 6-phosphofructokinase, and pyruvate kinase. Therefore, it is inferred that waterlogging stress induced an anoxic environment and resulted in the anaerobic respiration in peanut root cells to provide energy for life activities through the glycolysis process.

Other than the glycolysis process, the PPI interaction analysis of hub DAPs (Figure 7) also showed that waterlogging stress could lead to lactic acid and ethanol fermentation in treated plants. For example, the unique hub DAPs in XH08 were mainly related to lactic acid fermentation, such as L-lactate dehydrogenase, NAD^+^-dependent malic enzyme, aspartate aminotransferase, and glutamate dehydrogenase as well as the enzymes related to malic acid metabolism and the glyoxylic acid cycle. In agreement with these, the activities of ADH and LDH in treated cultivars were higher than the control groups (Figure 8), suggesting that other than glycolysis process, waterlogging stress induced anaerobic fermentation pathways, including lactic acid and alcohol fermentation (Wu et al., 2020). Lactic acid fermentation produces lactic acid, which would decrease cytosolic pH, thus inhibiting the LDH activity but activating ADH activity, which, as a result, lead to the transition from lactic acid fermentation to alcoholic fermentation (Dennis et al., 2000; An et al., 2016; Famiani et al., 2016; Wu et al., 2020; Pan et al., 2021). However, in waterlogging-tolerant plants, alcohol fermentation is more active than lactic acid fermentation (Dennis et al., 2000; Famiani et al., 2016; Wu et al., 2020; Pan et al., 2021), leading to slow accumulation of lactic acid and slow drop in cytosolic pH, and as a result, prolonged activity of LDH. These can be inferred from Figures 8A,B, 9, in which the ADH activity in XH08 was always higher than ZH4, while the LDH activity of XH08 extended 3 more days than ZH4 until 12 days after waterlogging treatment. It was reasonable to infer that the waterlogging-tolerant cultivar (XH08) had a more active ethanol fermentation activity, which slow down the accumulation of lactate, and therefore extended the lactic acid fermentation compared to the non-tolerant cultivar (ZH4).

Other than ADH and LDH, the MDH activity in XH08 was also more active than ZH4 throughout the waterlogging treatment process (Figure 8C). Coincidently, the expression of NAD^+^-dependent malic enzymes in hub DAP in mitochondria unique to XH08 was upregulated in the treated plants (Table 1). This coincided with the previous findings that NAD^+^-dependent malase could catalyze the decarboxylation of malate to pyruvate in the mitochondria, where the oxidation from malate to CO_2_ and NADH appears without going through glycolysis process to generate pyruvate (Famiani et al., 2016; Sun et al., 2019). Driscoll and Finan (1993) also found that NAD^+^-dependent malic enzymes produced by *Rhizobium meliloti* were necessary for symbiotic nitrogen fixation (Driscoll and Finan, 1993). Therefore, it is reasonable to infer that the waterlogging-tolerant cultivar might facilitate malic acid metabolism to accumulate pyruvate, producing energy to maintain normal physiological metabolism. It may also facilitate the symbiotic nitrogen fixation in soil microorganisms.

## Conclusion

Most previous studies mainly focused on the effects of waterlogging stress on plant morphology, physiology, dry matter accumulation and distribution, crop yield, and quality characteristics. Unveiling the molecular mechanisms under waterlogging stress will provide guidance for the future targeted peanut breeding. The current study found that ADH and MDH in XH08 were more active than ZH4, and the activity of LDH was prolonged in XH08 than ZH4, indicating that ethanol fermentation might be the primary anaerobic fermentation pathway in waterlogging-tolerant cultivar. In addition to anaerobic fermentation to provide energy, the waterlogging-tolerant cultivar might also rely on other metabolic pathways, such as malic acid metabolism to generate pyruvate to alleviate the energy shortage under waterlogging stress. This study enriched the current peanut genomics and proteomics resources, which will provide a foundation for future molecular breeding and genetic engineering in breeding for waterlogging-tolerant peanut cultivars.

## Data Availability Statement

The datasets “Peanut Root Waterlogging LC-MSMS”presented in this study are deposited to the ProteomeXchange Consortium via the PRIDE (Perez-Riverol et al., 2019) partner repository with the dataset identifier PXD027176.

## Author Contributions

DL and LL conceived and designed the study. JZ, HZ and NZ prepared samples for quantitative proteomics. WZ and JZ collected, curated the data, and wrote the manuscript. WZ did the data analysis. DL and LL were responsible for funding acquisition. WZ, HZ, ZL, DL, and LL revised the manuscript. All authors read and approved the manuscript.

## Conflict of Interest

The authors declare that the research was conducted in the absence of any commercial or financial relationships that could be construed as a potential conflict of interest.

## Publisher’s Note

All claims expressed in this article are solely those of the authors and do not necessarily represent those of their affiliated organizations, or those of the publisher, the editors and the reviewers. Any product that may be evaluated in this article, or claim that may be made by its manufacturer, is not guaranteed or endorsed by the publisher.

## Footnotes

^1^ http://www.maxquant.org
